# Epstein-Barr virus-associated acute pancreatitis: a clinical report and review of literature

**DOI:** 10.1186/s13052-022-01352-0

**Published:** 2022-09-05

**Authors:** Salvatore Accomando, Giulia Angela Restivo, Simona Scalzo, Melania Guardino, Giovanni Corsello, Mario Giuffrè

**Affiliations:** Department of Health Promotion, Mother and Child Care, Internal Medicine and Medical Specialties “G. D’Alessandro”, University of Palermo, Piazza Delle Cliniche, 2, 90127 Palermo, Italy

**Keywords:** Epstein-Barr virus (EBV), Pancreatitis, Children

## Abstract

**Background:**

Acute pancreatitis is a disorder of reversible inflammation of the pancreas. Only a few cases are related to infections and the most common pathogens are the viruses responsible for mumps, parotitis, and influenza. Epstein-Barr virus (EBV)-associated acute pancreatitis is a rare condition and it may occur in children and adults.

**Case presentation:**

A 3-year-old female was admitted to the “G. Di Cristina” Children's Hospital in Palermo for vomiting and abdominal pain. Laboratory investigations revealed elevated amylase and lipase, with normal liver function tests. Abdominal ultrasound demonstrated an enlarged pancreas, with hypoechogenic areas; no biliary lithiasis was observed. Infectious disease serology was positive for the presence of EBV VCA IgM and IgG. A diagnosis of EBV-associated acute pancreatitis was made. The patient was treated conservatively and recovered.

**Conclusions:**

Acute pancreatitis is rarely associated with EBV infection; a review of the English literature revealed only 10 pediatric and 6 adult cases. Patients with pancreatitis should always be evaluated for EBV serology, even in the absence of the typical clinical and hematological features of infectious mononucleosis. For these patients, good prognosis is generally expected.

## Background

Epstein-Barr virus (EBV) infection is a common viral infection, especially in children; it could have an asymptomatic course or may present as flu syndrome characterized by fever, malaise, headache, lymphadenitis, and pharyngitis [1]. EBV infection usually causes an acute self-limiting disease and resolves spontaneously in a few weeks; however, it may be responsible for various complications of the respiratory, cardiovascular, genitourinary, gastrointestinal, and nervous systems, ranging from minor to severe to life threatening. EBV also plays a significant role in the pathogenesis of autoimmune diseases and neoplasms [2].

As regards gastrointestinal manifestations, enlarged spleen and liver, with elevated transaminase levels, are the most common symptoms. Acute pancreatitis is a disorder of reversible inflammation of the pancreas. Only a few cases are related to infections and the most common pathogens are the viruses responsible for mumps, parotitis, and influenza. EBV-associated acute pancreatitis is a rare condition and it may occur in children and adults. Here we report on a 3-year-old girl with acute pancreatitis due to EBV infection; a review about this topic was also conducted thereafter.

### Case presentation

A 3-year-old female, previously in good health, was admitted to the “G. Di Cristina” Children's Hospital in Palermo for vomiting and abdominal pain. She did not complain of any other symptoms. Vital signs were normal. On physical examination, she appeared sick, with moderate epigastric tenderness. Laboratory investigations showed hemoglobin 13.8 g/dl, platelet 340,000/mm^3^, white blood cell count 16,600/mm^3^ (neutrophils 20%, monocytes 11%, and lymphocytes 69%), C-reactive protein 13,1 mg/dl (normal range < 0.5 mg/dl), aspartate aminotransferase 40 U/l, alanine aminotransferase 25 U/l, total bilirubin 0,11 mg/dl, amylase 913 U/l (normal range 30–100 U/l), and lipase 6450 U/l (normal range 3–32 U/l). Because of fair general conditions and elevated inflammatory markers, empirical antibiotic therapy with cefotaxime was started. Abdominal ultrasound revealed an enlarged pancreas, with hypoechogenic areas; no biliary lithiasis was observed. She had no past history of abdominal trauma, surgery or cholecystitis and any familial history of pancreatitis or gallstones was reported. She did not receive any medications known to cause pancreatitis. Serological tests for mumps, parotitis, rubella, EBV, cytomegalovirus, varicella-zoster virus, herpes simplex virus, coxsackie virus, and mycoplasma were all negative, except for the presence of EBV VCA IgM and IgG (EBNA IgG negative). Exudative pharyngotonsillitis, cervical lymphadenopathy, and hepatosplenomegaly were not present. A diagnosis of EBV-associated acute pancreatitis was made; the patient was treated conservatively, including fasting for three days until the resolution of vomiting, peripheral parenteral nutrition support for seven days, and pain management with acetaminophen. An abdominal ultrasound, performed after five days, did not show any complications and antibiotic therapy was discontinued. She improved clinically, lipase and amylase levels progressively decreased, and enteral feeding was gradually resumed. She was discharged home on the fifteen day of hospitalization.

## Discussion and conclusions

Acute pancreatitis is an inflammatory disorder of the pancreas; the incidence rate is 3–13 cases per 100,000 per year in the pediatric population [3, 4], and 5–60 cases per 100,000 persons per year in adulthood [5]. According to the American College of Gastroenterology guidelines, the diagnosis of acute pancreatitis is established by the presence of 2 of the 3 following criteria: (i) abdominal pain consistent with the disease, (ii) serum amylase and/or lipase greater than three times the upper limit of normal, and/or (iii) characteristic findings from abdominal imaging [6]. The most common etiology of acute pancreatitis is gallstones or microlithiasis; other causes include alcohol misuse, trauma, metabolic disorders (hypertriglyceridemia, hypercalcemia), infections (parotitis, mumps, influenza, herpes viruses, hepatitis viruses, coxsackie viruses, mycoplasma), systemic disease (hemolytic uremic syndrome, systemic lupus erythematosus, Henoch-Schönlein purpura, Kawasaki disease, inflammatory bowel diseases), and autoimmune pancreatitis [5, 7]. EBV infection is a rare cause of acute pancreatitis; the pathophysiology remains unclear: both direct viral infection and inflammatory process induced by the virus are plausible pathogenic mechanisms [8, 9].

A review of the English literature was performed: a PubMed search, using as keywords acute pancreatitis AND (EBV OR Epstein-Barr virus), revealed only 10 pediatric [8, 10–18] and 6 adult cases [9, 19–23]. As regards pediatric reports (Table I), median age and mean age was 12 and 11.8 years respectively (range 3–18), 36% were male and 64% were female. As regards pancreatitis symptoms, abdominal pain was described in all cases, vomiting in 55%, and nausea in 27%; eight patients (73%) also had mononucleosis symptoms, like fever, lymphadenitis, and pharyngitis. Amylase and/or lipase levels were increased up to three times the normal limit in 100% of the cases. In 5 children, there was evidence of acute pancreatitis on abdominal computerized tomography (CT), while only in our case, ultrasound (US) revealed an enlarged pancreas with a heterogeneous echotexture. The diagnosis of pancreatitis was confirmed in all patients, using the diagnostic criteria of the American College of Gastroenterology. Six children presented other complications related to EBV infection: the most common was cholestatic hepatitis (50%); cholecystitis, pneumonia, proctitis, portal vein thrombosis, and septic shock were also reported. Serological documentation for EBV infection was obtained in 10 cases, while in 1 child, the diagnosis was made clinically. All cases were treated with supportive care, that were fasting, intravenous fluids, parenteral nutrition, and/or pain management; in 1 patient, antibiotics and antivirals (meropenem, teicoplanin, and ganciclovir) were also used [17]. All children recovered.

**Table I:** Clinical data of pediatric cases with EBV-associated acute pancreatitis reported in literature and our case

Reference	Age/sex	Mononucleosis symptoms	Gastrointestinal symptoms	EBV diagnosis	Amylase-lipase	Imaging	Other complications	Therapy	Outcome
Wislocki et al. 1966 [10]	18y/M	Yes	Abdominal pain, vomiting	Heterophil antibody	480 U/l-NA	NA	No	Fasting, intravenous fluids, analgesics	Recovered
Hedstrom et al. 1976 [11]	12y/F	Yes	Abdominal pain, nausea	Clinical	8700 U/l-NA	NA	No	Symptomatic	Recovered
Werbitt et al. 1980 [12]	16y/M	Yes	Abdominal pain, nausea, vomiting	VCA positivity	378 U/l-NA	CT: no pancreatic abnormality	No	Not available	Recovered
Koutras et al. 1983 [13]	8y/F	Yes	Abdominal pain, vomiting	VCA IgM positivity	300–180 U/l	NA	Cholestatic hepatitis, proctitis	Symptomatic	Recovered
Khawcharoenporn et al. 2008 [14]	18y/F	Yes	Abdominal pain	VCA IgM positivity	620–659 U/l	CT: edematous pancreas	Cholecystitis, septic shock, DIC	Symptomatic	Recovered
Kang et al. 2013 [15]	11y/F	No	Abdominal pain, vomiting	VCA IgM positivity	4010–4941 U/l	CT: edematous pancreas, peripancreatic fluid accumulation	Cholestatic hepatitis	Fasting, parenteral nutrition	Recovered
López-Ibáñez et al. 2013 [16]	15y/M	Yes	Abdominal pain	Heterophil antibody	1251 U/I-NA	CT: globular pancreas, hepatosplenomegaly, ascites	No	Not available	Recovered
Galzerano et al. 2014 [17]	3y/F	No	Abdominal pain	VCA IgM positivity	3880 U/l-NA	CT: enlargement of the pancreatic head	Bilateral pneumonia, portal vein thrombosis, septic shock	Fasting, parenteral nutrition, meropenem, teicoplanin, ganciclovir	Recovered
Narchi et al. 2014 [18]	8y/M	Yes	Abdominal pain, vomiting	VCA IgM positivity	80–1000 U/l	MRI: not visible pancreas	Cholestatic hepatitis, cholecystitis	Fasting, intravenous fluids	Recovered
Hammami et al. 2019 [8]	18y/F	Yes	Abdominal pain, nausea	VCA IgM positivity	327–2016 U/l	CT: signs of acute pancreatitis	Hepatitis	Symptomatic	Recovered
Our case	3y/F	No	Abdominal pain, vomiting	VCA IgM positivity	913–6450 U/l	US: enlargement of the pancreas	No	Fasting, parenteral nutrition, analgesics	Recovered

Abbreviations**:** *y* years, *M* Male, *F* Female, *NA* Not available, *DIC* Disseminated intravascular coagulopathy

As regards adult patients (Table II), EBV-associated acute pancreatitis affects mainly young adults (range 21–45 years), with a slight female predominance (66%). All cases presented abdominal pain, associated sometimes with nausea, fever, and vomiting. In 3 patients (50%), signs and symptoms related to infectious mononucleosis were also observed. The diagnosis of EBV infection was made by positive serology in 5 patients; also in 2 cases, serum EBV-DNA was detected. Abdominal CT was executed in 5 patients, revealing signs of acute pancreatitis, such as enlarged and edematous pancreas; in 1 case, areas of necrosis were also noticed. All patients except one had complications related to systemic EBV infection, revealing a more severe clinical course in adults than children. The reported complications are hepatitis with or without cholestasis, gastritis, pneumonia with pleural effusion, ascites, pericardial effusion, autoimmune hemolytic anemia, and multi-organ failure. The patients were treated with symptomatic therapy; antibiotics, antivirals, and steroids were also administered in critical cases. All patients except one fully recovered.

**Table II:** Clinical data of adult cases with EBV-associated acute pancreatitis reported in literature

Reference	Age/sex	Mononucleosis symptoms	Gastrointestinal symptoms	EBV diagnosis	Amylase-lipase	Imaging	Other complications	Therapy	Outcome
Jahann et al. 2012 [19]	22y/M	Yes	Abdominal pain	VCA IgM positivity	330–2300 U/l	NR	No	Symptomatic	Recovered
Cook et al. 2015 [20]	25y/M	No	Abdominal pain, nausea, fever	VCA IgM positivity	NA-429 U/l	CT: pancreatic edema	Cholestatic hepatitis, pleural effusions, ascites	Fasting, parenteral nutrition, analgesics	Recovered
Singh et al. 2015 [21]	21y/F	Yes	Abdominal pain, vomiting, nausea	VCA IgM positivity	NA-4301 U/l	CT: pancreatic edema	Autoimmune hemolytic anemia	Symptomatic, prednisone	Recovered
Zhu et al. 2017 [9]	35y/F	Yes	Abdominal pain, vomiting	VCA IgM positivity	1300–1450 U/l	CT: pancreatic edema	Hepatitis, pneumonia	Fasting, parenteral nutrition, amoxicillin-clavulanate, acyclovir	Recovered
Fiani et al. 2021 [22]	35y/F	No	Abdominal pain, fever	VCA IgM and IgG positivity, serum EBV DNA	129/408 U/l	CT: enlargement of the pancreas	Cholestatic hepatitis, pneumonia with pleural effusions	Symptomatic, linezolid, meropenem, oseltamivir, acyclovir, methylprednisolone	Recovered
Huang et al. 2021 [23]	45y/F	No	Abdominal pain	Serum EBV DNA	Increased up to three times the normal limit	CT: pancreatic necrosis	Pericardial and pleural effusions, gastritis, MOF	Symptomatic	Dead

Abbreviations: *y* years, *M* Male, *F* Female, *NA* Not available, *NR* Not reported, *MOF* Multi-organ failure

In conclusion, EBV infection is characterized by clinical heterogeneity; multiple organs could be involved, also the pancreas, both in children and young adults. Active surveillance is needed for prompt diagnosis and early treatment. In patients with signs and symptoms of acute pancreatitis, EBV infection should always be considered, even in the absence of the typical clinical and hematological features of infectious mononucleosis. Generally, EBV-associated acute pancreatitis is characterized by a favorable prognosis, with a spontaneous resolution.

## Data Availability

The datasets used and analyzed during the current study are available from the corresponding author on reasonable request.

## Abbreviations

EBV: Epstein-barr virus
CT: Computerized tomography
US: Ultrasound
